# Small-Angle Neutron Scattering Instrument Concepts for Second Target Station of SNS

**DOI:** 10.1063/4.0000933

**Published:** 2025-10-27

**Authors:** Shuo Qian

**Affiliations:** Oak Ridge National Laboratory, Oak Ridge, TN, USA

## Abstract

Small-Angle Neutron Scattering (SANS) is a powerful and popular technique widely used for structural characterization in many science and engineering fields. The Second Target Station (STS) of the Spallation Neutron Source (SNS) at Oak Ridge National Laboratory (ORNL) has been specifically designed to provide a broad bandwidth, high-brightness cold neutron source that is ideally suited for SANS instruments. Along with the advancements in neutron optics and computational algorithms, STS maximizes the potential of future SANS instruments at ORNL to provide the user community with unprecedented capabilities. For example, users will be able to perform experiments with ten times less sample, simultaneously measure phenomena at both atomic and mesoscopic scales, and investigate sub-second kinetic processes under in situ/operando conditions.

In this presentation, the current instruments under development are introduced as follows:

**CENTAUR:** Already in the preliminary design phase, this is the most mature SANS instrument currently planned. It is a multifunctional workhorse with simultaneous wide-angle (WANS) and diffraction coverage. Featuring a variable octagon guide system, a statistical chopper for inelastic neutron suppression, and generous detector coverage, it serves a broad community—from soft matter, biology, chemistry, and materials science to quantum sciences.

**FocuSANS:** A concept in development, FocuSANS is a very high-throughput/sub-second kinetics SANS/WANS instrument with focusing optics for millimeter-sized samples and moderate instrumental resolution (due to the smearing effect and limited low-Q coverage). It prioritizes high neutron flux and fast data acquisition, making it suitable for time-resolved studies of dynamic processes on sub-second timescales, experiments on very weakly scattering samples or limited sample quantities, and high-throughput characterization of industrial materials.

**Lab-On-SANS:** Another concept in development, this mission-specific instrument is designed to rapidly measure material microstructure during real-world processing conditions, such as additive manufacturing, injection molding, or in situ polymerization. Featuring a less configurable optical guide system, it provides relatively high resolution and broad Q coverage with a modular sample area that can integrate different laboratory setups as required by users.

**Auto-SANS:** Also in development, this instrument will be highly integrated with Artificial Intelligence (AI). It will operate autonomously with robotic systems to enable self-driving sample synthesis, preparation, characterization, and analysis directly at the instrument. Multi-modal characterizations—such as X-ray and light scattering—will also be incorporated to provide holistic information that aids SANS experiments and achieves optimized sample conditions.

